# Music as a Gateway to Men’s Mental Health: A Theoretical Framework for Masculinity-Congruent Intervention

**DOI:** 10.1177/15579883261481861

**Published:** 2026-09-07

**Authors:** Jared Kyle Sieusahai

**Affiliations:** 1 Faculty of Medicine and Dentistry, 3158 University of Alberta, Edmonton, AB, Canada

**Keywords:** male mental health, masculinity < gender issues and sexual orientation, help seeking, gateway mechanisms, veterans

## Abstract

Men consistently underutilize formal mental health services due to masculinity norms that frame help-seeking as incompatible with masculine identity. This opinion piece proposes the gateway mechanism as a theoretical framework for understanding why music-based interventions may be particularly effective for reaching men who resist conventional services. The gateway mechanism is defined as the process by which a masculinity-congruent, non-clinical activity may create the psychological safety, relational trust, and reduced self-stigma through which men gradually move toward emotional disclosure and eventual help-seeking. Masculinity-congruent refers to activities and framings aligned with values men associate with masculine identity, including competence, agency, and camaraderie, rather than vulnerability or therapeutic dependency. The framework specifies four sequential stages: initial engagement through intrinsic musical appeal; development of psychological safety; emotional expression through and about music; and disclosure and help-seeking that would not otherwise have occurred. It draws on help-seeking theory, self-stigma frameworks, gender role strain theory, and psychological safety literature. Converging evidence from veteran music therapy programs, the Men’s Sheds literature, and culturally specific music therapy research supports the framework’s predictions. Alternative mechanisms, including the possibility that music functions primarily as a hook into protective social processes such as peer support, camaraderie, and shared identity, are acknowledged. The gateway mechanism is distinguished from health by stealth, gender-sensitive engagement, and sport-as-gateway models by its specification of the sequential psychological process through which musical engagement may produce conditions for emotional opening. Future research should employ longitudinal designs, incorporate masculinity norms instruments, and test three hypotheses arising from the framework of Music as a Gateway to Men’s Mental Health: A Theoretical Framework for Masculinity-Congruent Intervention Keywords: male mental health; music therapy; masculinity norms; help-seeking; gateway mechanism; veterans; community-based intervention.

## Introduction

Men bear a disproportionate burden of undertreated mental health problems globally, yet consistently underutilize formal psychological services across the life course (Bilsker et al., 2018; Oliffe et al., 2021).

In Canada, men account for approximately 75% of suicide deaths (Statistics Canada, 2023), and systematic reviews consistently find that conformity to traditional masculine norms including self-reliance, emotional stoicism, and strength is the primary driver of men’s avoidance of help-seeking (Mokhwelepa & Sumbane, 2025; Seidler et al., 2016).

The structural problem is well-established: men do not access services designed for them, and designing better services has not reliably solved this problem.

This opinion piece proposes the gateway mechanism as a theoretical framework for understanding why music therapy and music-based interventions may be particularly effective for reaching men who resist conventional mental health services. Throughout this paper, a distinction is maintained between music therapy, which involves structured therapeutic interventions delivered by credentialed music therapists with specific clinical goals, and music-based interventions, which refer to non-clinical, group-based musical activities organized around shared participation. Both may instantiate the gateway mechanism, though through partially distinct pathways.

The gateway mechanism is defined here as the process by which a masculinity-congruent, non-clinical activity creates the psychological safety, relational trust, and reduced self-stigma through which men gradually move toward emotional disclosure and eventual help-seeking. Masculinity-congruent refers to activities, settings, and framings that align with the values, social norms, and identity frameworks that men in a given cultural context associate with masculinity, including competence, agency, camaraderie, and purposeful activity, rather than vulnerability, emotional need, or therapeutic dependency.

The framework draws on converging evidence from music therapy, men’s mental health, and community-based intervention research to propose a theoretically grounded and empirically supported account of how music reaches men that other approaches do not.

The gateway mechanism is distinct from, though related to, existing concepts in men’s health. Health by stealth describes the embedding of health-relevant activity within masculine-coded practical engagement (Cordier & Wilson, 2014; Wilson & Cordier, 2013), as documented extensively in the Men’s Sheds literature. Gender-sensitive engagement describes the adaptation of service delivery to masculine preferences and communication styles (Seidler et al., 2020). Activity-based approaches to men’s help-seeking describe the use of shared doing as a vehicle for social connection and incidental health conversation (McEvoy et al., 2023).

The gateway mechanism builds on these concepts by specifying the sequential psychological process through which initial activity-based engagement produces the conditions for emotional opening and help-seeking, and by proposing music as a specific activity with particular gateway properties grounded in both its cultural significance in masculine identity and its neurobiological capacity for emotional engagement.

The four-stage sequence draws on several established theoretical traditions. Help-seeking theory identifies perceived stigma and masculine role conflict as the primary barriers to formal service use; the gateway mechanism addresses these barriers by removing the need for help-seeking identification altogether. Self-stigma frameworks suggest that men who have internalised masculine norms experience shame in acknowledging mental health need, and that interventions which provide an alternative identity-congruent rationale for participation reduce this barrier. Gender role strain theory frames men’s help-seeking resistance not as individual pathology but as a rational response to socialisation; the gateway mechanism works with rather than against this socialisation. Psychological safety literature suggests that group cohesion and trust must precede emotional disclosure; the four-stage sequence is consistent with this, proposing that collective musical engagement builds the psychological safety that enables subsequent emotional openness. Behaviour change models, particularly those emphasising environmental restructuring and habit formation, support the argument that sustained exposure to prosocial, emotionally activating contexts through repeated musical participation can gradually shift men’s relationship with emotional expression. 

## The Gateway Mechanism: Definition and Stages

The gateway mechanism operates through four sequential stages, each of which is supported by empirical evidence from music therapy and related literatures. The first stage is initial engagement through intrinsic appeal. Men engage with music-based programs not because they identify as needing mental health support but because music itself is appealing: they want to play an instrument, participate in a group drumming session, listen to music that resonates with their identity, or engage with a musical format that carries cultural meaning for them.

This engagement does not require acknowledgment of need, identification as a patient, or acceptance of clinical framing. A veteran who would decline referral to group therapy attends a music program because he values the music, not because he seeks help (Drozd, 2022; Vaudreuil et al., 2022).

The second stage is development of psychological safety within the musical context. Sustained participation in a music-based group or therapeutic relationship gradually produces the relational conditions within which emotional disclosure becomes possible.

The mechanisms here are multiple: musical synchrony may produce states of interpersonal attunement and trust (Roth, 2025); shared activity creates a context of mutual investment that reduces the individual exposure of one-on- one clinical encounters; the non-verbal dimension of musical engagement provides an alternative channel for emotional expression that does not require verbal articulation of distress. Psychological safety in this context means specifically that the costs of emotional expression are reduced to a level that masculine norms do not prohibit.

The third stage is emotional expression through and about music. Within the safety of the musical context, men begin to express emotional material that they would not articulate in formal clinical settings.

This may take the form of song selection that reflects emotional states, lyrical themes in songwriting that address personal experiences, verbal conversation about what the music means or has meant in their lives, or physical and physiological emotional responses to music that bypass the cognitive suppression that masculine norms otherwise produce (Adeyola & Allen, 2025; Huang & Chen, 2021).

The music may function as a licensed vehicle for emotional expression: a culturally sanctioned context within which emotional responses are permitted and even expected, reducing the self-stigma that prevents men from expressing distress in clinical contexts (Hogan, 1999). The fourth stage is disclosure and help-seeking that would not otherwise have occurred. Men who have progressed through the first three stages have developed the relational trust, reduced self-stigma, and emotional vocabulary that enable them to acknowledge distress and seek support, either within the music therapy relationship itself or by accessing other formal services.

The gateway hypothesis specifically predicts that music-based engagement may lead toward rather than away from formal help-seeking: the trust and openness produced by musical participation may create conditions under which men become more likely to engage with the broader mental health system, not less.

## Evidence for Music Specifically

The gateway mechanism is supported by converging evidence across several research traditions, none of which was designed to test the hypothesis directly but each of which is consistent with its predictions.

The most direct experimental support comes from the Boys Do Cry randomized controlled trial, which tested the effects of a music video specifically designed to encourage men to seek help for mental health challenges (Nicholas et al., 2025). The trial found that exposure to the music video produced significant improvements in help-seeking intentions and attitudes among men, providing direct experimental evidence that music-based content may shift the masculine norms that prevent men from seeking support. Crucially, the mechanism was not simply exposure to a mental health message: a control condition with equivalent mental health content in non-musical format produced weaker effects, suggesting that the musical format itself may have contributed to the gateway effect.

The veteran music therapy literature provides the strongest naturalistic evidence for the gateway mechanism. Veterans represent a population with pronounced masculine norms, high mental health burden, and well-documented resistance to formal psychological services (Drozd, 2022; Uomoto et al., 2025).

The consistent finding across multiple studies and program evaluations that this population engages with music therapy at substantially higher rates than with conventional mental health services is itself theoretically significant (Powers et al., 2012; Vaudreuil et al., 2022). A programmatic evaluation of a music therapist-led community program for veterans in supported housing found that participants consistently preferred rock, country, blues, and folk music, and most frequently engaged through singing and instrument play (Gooding et al., 2025).

These genre and modality preferences are not incidental: they reflect masculine-coded musical formats that fit within a framework of active doing, skill-based participation, and cultural identity that passive, receptive, or verbal clinical formats do not provide. The engagement patterns documented in this evaluation are precisely what the gateway mechanism predicts: men engaging through music formats that align with their masculine identity, in a context that does not require them to identify as help-seekers. The Men’s Sheds literature provides parallel evidence for the health by stealth mechanism that the gateway framework extends. Mixed-methods systematic reviews consistently find that Men’s Shed participation is associated with reduced social isolation, improved mental health, and increased health literacy, through the mechanism of activity-based engagement that embeds health-relevant conversation within practical work (Cordier & Wilson, 2014; Wilson & Cordier, 2013). A path-model survey of 333 Shed members found that psychological safety within the Shed predicted engagement, engagement predicted expanded social networks and meaning in life, and both predicted improved wellbeing and lower loneliness (McEvoy et al., 2023).

This sequential structure mirrors the gateway mechanism’s stages and suggests that the framework generalizes beyond music to other masculinity-congruent activity-based interventions. Qualitative evidence from rap music therapy with Black men provides culturally specific support for the gateway mechanism’s account of how musical format interacts with masculine identity (Adeyola & Allen, 2025). This research found that the rap format created a culturally congruent context within which Black men could express anger, depression, and vulnerability that they would not articulate in predominantly white or conventionally clinical therapeutic spaces.

The finding that cultural specificity in music selection and format was a prerequisite rather than an enhancement for therapeutic engagement directly supports the gateway mechanism’s prediction that the masculinity-congruence of the musical activity determines its gateway effectiveness. Null and qualified findings deserve acknowledgment. Not all music-based programs produce gateway effects, and the existing evidence does not support the conclusion that music therapy is universally effective for men. Studies vary substantially in engagement rates, and the conditions that maximize gateway effects have not been systematically investigated.

The gateway mechanism is a hypothesis supported by converging evidence rather than an established empirical law, and it requires prospective longitudinal testing to confirm the sequential predictions it makes. Several additional limitations must be acknowledged. First, the evidence base draws predominantly from Western, English-language contexts; the applicability of the gateway mechanism framework to non-Western cultural contexts, where masculine norms, music’s cultural valence, and community infrastructure differ substantially, remains untested. Second, substantial heterogeneity among men and masculinities means that the framework may not apply uniformly across age groups, cultural backgrounds, sexual orientations, and socioeconomic contexts; men who do not identify with music-based activities or for whom other activity-based gateways are more culturally salient may not be reached by this model. Third, potential selection effects must be acknowledged: men who self-select into community music programs may already be more open to peer connection and emotional engagement than the most disengaged men who constitute the hardest-to- reach population. Fourth, the proposed sequential mechanism has not been directly tested longitudinally; the causal direction of the relationship between musical engagement and improved mental health outcomes cannot be established from cross-sectional or qualitative evidence alone.

## Distinguishing the Gateway Mechanism from Related Concepts

The gateway mechanism is distinguished from health by stealth primarily by its specificity about the psychological process through which activity-based engagement produces health outcomes. It is important to situate the framework relative to existing activity-based gateway models. Sport-as- gateway research, including Duffell et al.'s (Duffell et al., 2025) qualitative study of men in a professional football club community programme, has consistently found that men engage initially through the sport itself, but that sustained engagement and health benefits are driven by camaraderie, friendship, peer support, belonging, and social connection rather than the sport per se. Football was the hook; the social environment became the sustaining mechanism. The gateway mechanism proposed here is fully consistent with this finding and does not claim otherwise.

What is novel is not the gateway concept itself but its music-specific instantiation: the argument that music, among activity-based gateways, carries a particular combination of cultural salience to masculine identity and neurobiological capacity for producing emotional openness and interpersonal synchrony that may make it especially effective as a gateway mechanism, and the specification of the sequential psychological stages through which this operates. Health by stealth describes a structural feature of effective men’s health programs, namely that health benefits are produced by and through activities that men value for their own sake.

The gateway mechanism adds a sequential account of how this works psychologically: the activity produces specific psychological changes, reduced self-stigma, increased psychological safety, licensed emotional expression, that enable help-seeking that would not otherwise occur. The gateway mechanism is distinguished from gender-sensitive engagement by its focus on the entry point rather than the service adaptation. Gender-sensitive engagement describes how existing services can be modified to be more acceptable to men who are already engaged. The gateway mechanism addresses the prior problem: how to reach men who would not engage with any service regardless of how it is adapted. The gateway is not a modified door into an existing building but a separate entry point that leads eventually to the building.

The gateway mechanism is distinguished from activity-based approaches to men’s help-seeking by its specification of music as an activity with particular gateway properties beyond those of other activities. Music’s gateway effectiveness derives from its neurobiological capacity to engage emotional and social brain systems independently of verbal cognitive processing (Koelsch & Bradt, 2025), its role as a licensed vehicle for emotional expression in masculine cultural contexts, its capacity for cultural specificity in format and genre, and its dual function as both a social activity and an emotional experience.

These properties are not shared by all activities that might function as health by stealth entry points.

## Clinical and Research Implications

**Figure 1.**
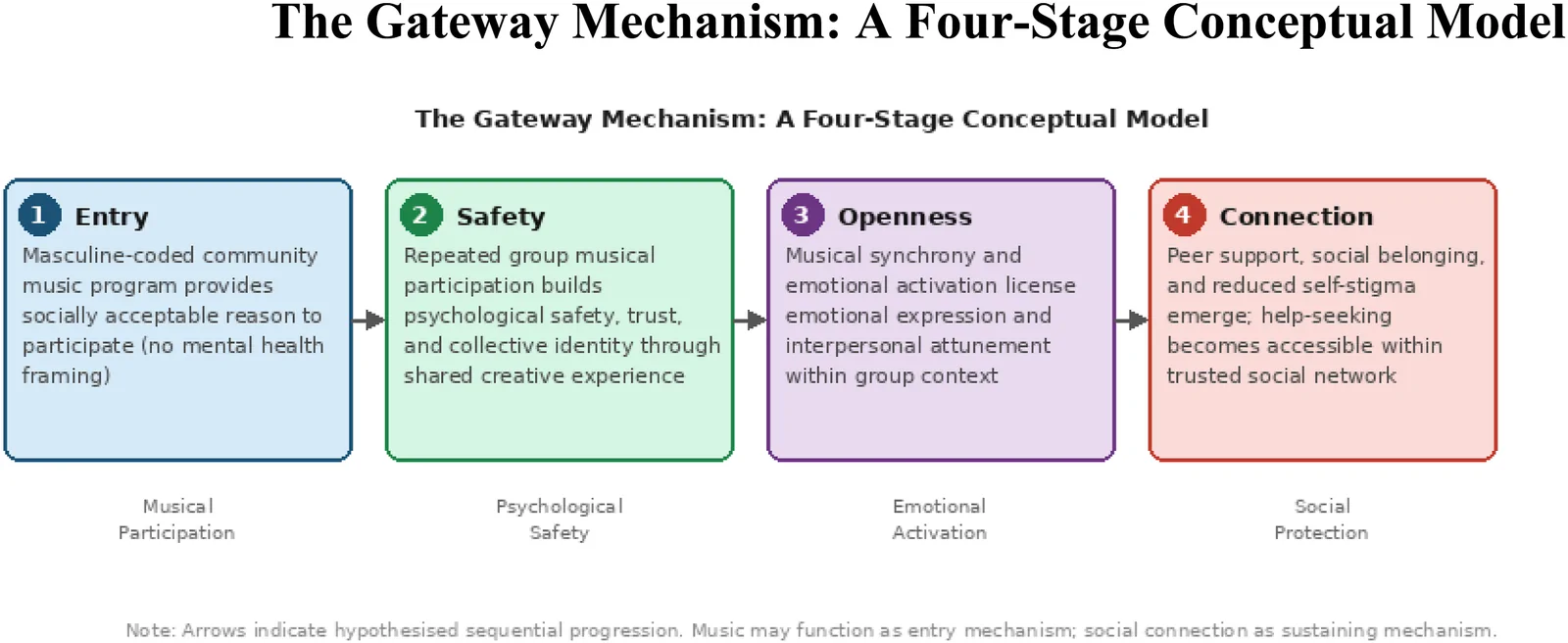
The Gateway Mechanism: A Four-Stage Conceptual Model. *Note.* Arrows indicate hypothesised sequential progression. Music may function as entry mechanism; social connection as sustaining mechanism

The gateway mechanism framework has direct implications for how music therapy programs for men should be designed, positioned, and evaluated. Programs should be explicitly framed around musical participation rather than mental health support, using the music itself as the primary stated purpose and allowing health-relevant engagement to develop within that context. Genre and modality should be selected to maximize alignment with participants’ masculine cultural identity, recognizing that this will vary substantially across veteran, working-class, Black, Indigenous, and other male populations. Group formats that create conditions for musical synchrony and peer social connection should be prioritized over individual receptive formats for populations with high masculine norm conformity. Future research should employ longitudinal designs that track men’s movement from initial music- based engagement through the stages of the gateway mechanism to formal help-seeking over time, to test the sequential predictions of the framework directly. Sex-disaggregated reporting should be mandatory in all music therapy trials. Validated masculinity norms instruments including the Conformity to Masculine Norms Inventory (CMNI-46) (Mahalik et al., 2003) should be incorporated alongside mental health outcome measures in music therapy program evaluations. Direct comparison of music-based entry point programs with standard mental health outreach strategies for men would establish the incremental value of the gateway approach Figure 1.

The Gateway Mechanism: A Four-Stage Conceptual Model.

## Conclusion

The gateway mechanism framework provides a theoretically grounded and empirically supported account of why music therapy and music-based interventions may be particularly effective for reaching men who resist conventional mental health services. By specifying the sequential psychological process through which masculinity-congruent musical engagement produces the conditions for emotional disclosure and help-seeking, the framework extends existing concepts of health by stealth and gender-sensitive engagement with direct implications for clinical program design and research priorities. Prospective longitudinal research testing the gateway mechanism directly is needed to establish it on the robust empirical footing that its clinical implications warrant. Three testable hypotheses follow from the framework: (1) men who participate in community music programs framed around musical participation rather than mental health will show greater sustained engagement than men in equivalent mental-health-framed programs; (2) the pathway from musical engagement to improved mental health outcomes will be mediated by social connectedness, peer support, and sense of belonging rather than by music-specific neurobiological effects alone; and (3) men in music-based gateway programs will show higher rates of subsequent engagement with formal mental health services than comparable men without program access, consistent with the gateway mechanism’s help-seeking premise.
